# Genome-wide CRISPR/Cas9 library screening identified ATM signaling network genes as critical drivers for resistance to ATR inhibition in soft-tissue sarcomas: synthetic lethality and therapeutic implications

**DOI:** 10.1186/s40164-023-00416-z

**Published:** 2023-05-31

**Authors:** M Spalato-Ceruso, A Laroche-Clary, R Perret, Y Valverde, V Chaire, Marie-Alix Derieppe, V Velasco, A Bourdon, A Italiano

**Affiliations:** 1 Sarcoma Unit, Institut Bergonié, 229 cours de l’Argonne, Bordeaux, 33000 France; 2 INSERM, U1312, Bordeaux, France; 3 Department of Pathology, Institut Bergonié, Bordeaux, France; 4 University of Bordeaux, Bordeaux, France; 5 Bioinformatics, Data and Digital Health Departement, Insitut Bergonié, Bordeaux, France

**Keywords:** ATR, ATM, CRISPR/Cas9, Synthetic lethality

## Abstract

Soft-tissue sarcoma (STS) are a heterogeneous group of rare tumors with different biological behavior that are fatal in more than 40% of cases, due to their metastatic evolution and inadequate treatment options. ATR inhibition already showed an activity, even if modest, in broad pre-clinical models of STS. By using genome-wide CRISPR/Cas9 library screening, we identified ATM signaling network genes as critical drivers for resistance to the specific ATR inhibitor AZD6738. The role of such genes in resistance to AZD6738 was confirmed by using CRISPR/Cas9 knockout models. More strikingly, the ATM inhibitor AZD0156 works synergistically with AZD6738 in vitro and abolishes STS growth in vivo in our models of most frequent histotypes (such as dedifferentiated liposarcoma, leiomyosarcoma, and undifferentiated pleomorphic sarcoma among others). Moreover, the combination of AZD6738 and AZD0156 induced significantly higher levels of DNA damage than either drug used as single agent alone. In summary, our results demonstrate that targeting ATM is an effective approach to overcome resistance to ATR inhibition in different STS subtypes, including the most frequent histologies.

**Supplementary Information:**

The online version contains supplementary material available at 10.1186/s40164-023-00416-z.

## To the editor

Soft tissue sarcoma (STS) are a heterogeneous group of rare tumors including more than 70 subtypes with distinct clinical and biological features [1]. Surgical resection represents the cornerstone of treatment of all patients with localized STS. However, up to 40% of patients who underwent optimal surgery will develop metastatic disease [2]. Only few drugs including doxorubicin, or gemcitabine have shown activity in the advanced setting and the median overall survival has only slightly improved in the last 20 years from 12 months to 18 months [3]. There is a crucial need for new and effective drugs for patients with advanced STS.

Gene expression profiling of a large cohort of STS allowed the identification and validation of a 67-gene signature of chromosome instability named CINSARC (for genome Complexity INdex in SARComas), which is the most significant predictor of metastasis-free survival in these tumors [4]. Interestingly, many of the identified genes encode for proteins involved in DNA damage repair.

Moreover, a recent international study has shown that germline variants in several genes encoding proteins involved in DNA repair, such as BRCA2, ATM, ATR, and ERCC2, contributed significantly to sarcoma risk [5].

ATR plays a crucial role in maintaining genomic integrity by responding to a large spectrum of DNA damages, including double strand breaks (DSBs) that interfere with replication. We have previously shown that ATR inhibition has an activity, even if modest, in a large number of pre-clinical models of STS [6]. To maximize the effectiveness of ATR inhibitors (ATRi), combinations with agents targeting mechanisms of resistance to ATR inhibition should be identified.

Here, we propose to investigate the mechanisms of resistance to ATR inhibition in STS by using a genome-wide negative CRISPR screening of a resistant STS cell line, and to validate treatment strategies to restore sensitivity (see Additional file 1: Methods).

To examine the antitumor effect of ATR inhibition on STS, a panel of 7 STS cell lines (well differentiated and dedifferentiated liposarcoma, leiomyosarcoma, myxofibrosarcoma, and extraskeletal osteosarcoma) were plated and treated with increasing concentrations of the specific ATRi AZD7638 for 72 h. We showed that AZD7638 suppressed the viability of 5 of them, encompassing several histological subtypes, with IC_50_ values ranging from 1.03 to 4.6µM (Supplementary Fig. 1). However, the two leiomyosarcoma cell lines displayed primary resistance to AZD7638: IB112: IC_50_ 12.5µM; IB136: IC_50_ not determined (Supplementary Fig. 1).

**Fig. 1:**
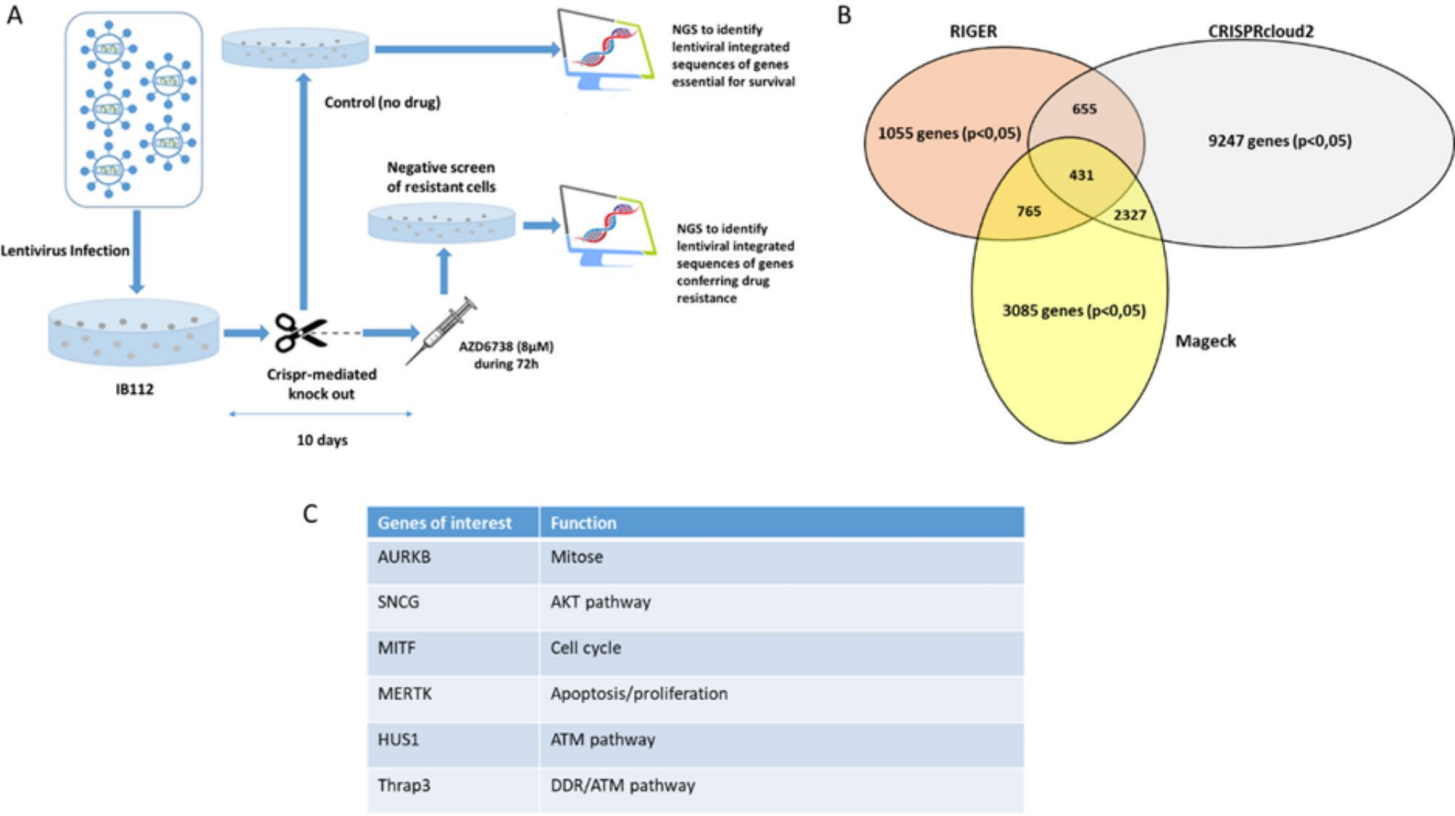
** (A)** genome-wide CRISPR/Cas9 negative selection, identification of lost guides corresponding to genes implicated in the resistance to AZD6738 compound. Briefly, for the CRISPR screen with the library GeCKO, 240 millions of IB112 were infected at a MOI = 0.3 and cells were selected during 3 days with puromycin, after selection we let cells grow during approximately one week. For the resistance screen, 30 × 10^6^ cells were treated by DMSO and 30 × 10^6^ cells were treated by 8µM of ATRi. DNA was extracted for each condition, sequenced and compared. **(B)** Sequences were analyzed with 3 different bioinformatics tools: RIGER, CRISPRcloud2 and Mageck. **(C)** Genes of interest identified with at least two bioinformatics analysis

Therefore, we decided to perform a CRISPR/Cas9-negative selection screening on one of the two resistant cell lines to identify genes involved in ATRi resistance. For this purpose, we performed the CRISPR/Cas9 screening on the IB112, one of the most resistant cell lines to AZD6738. We applied a negative selection screening, treating cells with suboptimal dose of AZD6738, to select the knockout cells most sensitive to the treatment, (see Suppl. Materials and Methods). Thus, among the significant genes identified, we found genes involved in DNA Damage Response (DDR) pathway and specifically Ataxia Teleangectasia Mutated (ATM) pathway (such as THRAP3 and HUS1), mitosis, apoptosis, and cell cycle (such as AURKB, MERTK and MITF) (Fig. 1).

Among these candidates, we selected THRAP3 for further validation given its statistically significant representation, and the fact that its suppression has been associated with defective DNA repair through the impairment of the mRNA splicing and export of transcripts of several key DDR proteins, including the ATM kinase [7] (Supplementary Fig. 2). MTT assay confirmed that sgRNA targeting THRAP3 strongly sensitized IB112 cells to AZD6738. Cell cycle experiments revealed that silencing of THRAP3 suppressed the S phase accumulation induced by AZD6738 in IB112 cells (Supplementary Fig. 3).

These data confirms that THRAP3 is involved in resistance to ATR inhibition in leiomyosarcoma cells. Given that THRAP3 is not targetable pharmacologically and given its role in ATM regulation, we decided to evaluate if the combination of a clinical compound targeting ATM could be synergistic with AZD6738 in our panel of STS. We used the selective and potent ATM kinase inhibitor (ATMi) AZD0156 in combination with AZD6738. The Chou-Talalay score showed that the ATRi/ATMi combination is synergistic or additive in all seven STS cell lines (Supplementary Fig. 4). Increased apoptosis was also observed with the combination treatment in comparison with each drug used as single agent (Supplementary Fig. 5). Having found that targeting ATM with AZD0156 can sensitize STS cells to AZD6738, we next investigated the DDR signaling pathways activated by AZD6738 in the presence or absence of ATM function. We found that AZD6738 reduces the level of phosphorylated CHK1 (pCHK1) and upregulates pATM. On the opposite, AZD0156 upregulated pCHK1 and reduced the level of pATM (Supplementary Fig. 6). These results are suggestive of a compensatory effect and cross-talk between the ATM and ATR-dependent checkpoint response pathways. The combination of AZD6738 and AZD0156 synergistically reduced the levels of pCHK1 and of pATM in all the cell lines analyzed including the cell lines resistant to AZD6738 (IB112 and IB136) (Supplementary Fig. 6).

**Fig. 2:**
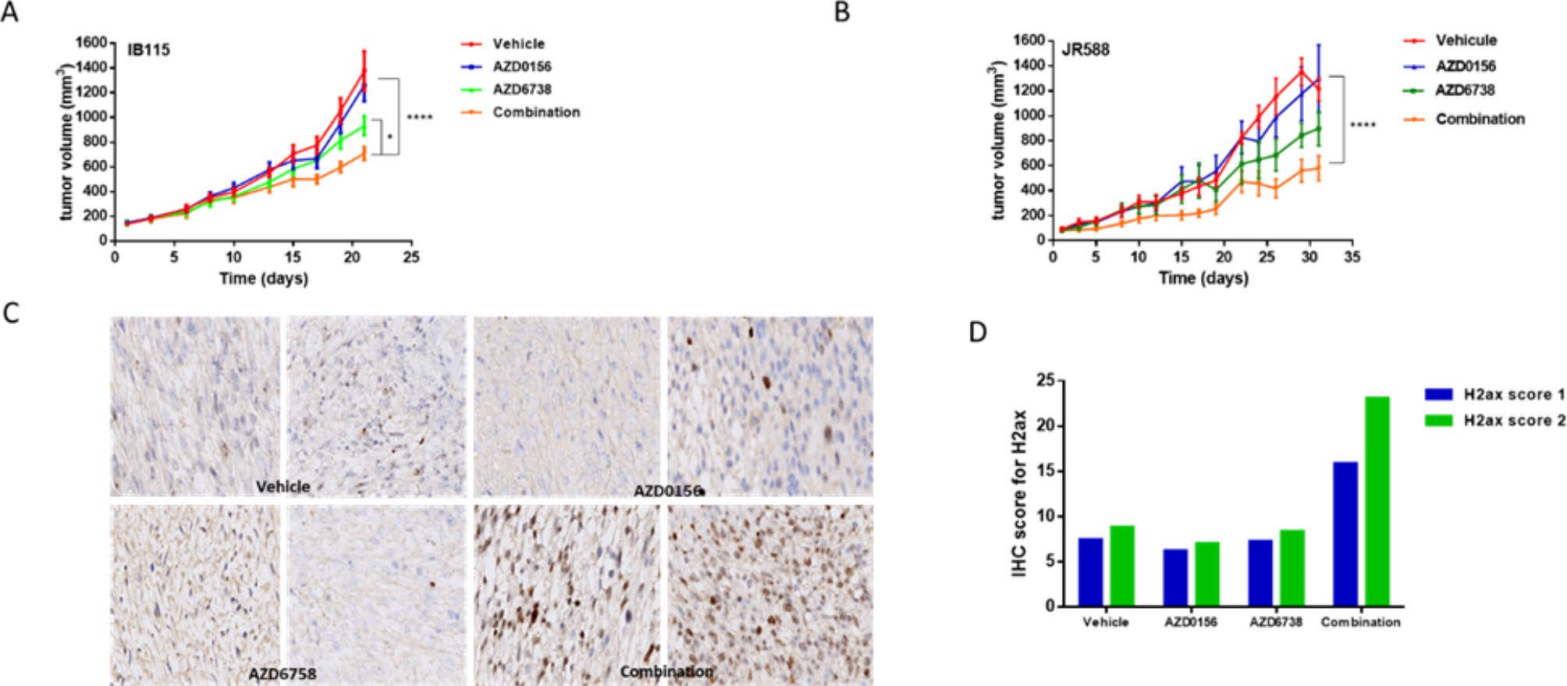
In vivo analysis of combined ATR (AZD6738) and ATM (AZD0156) inhibition in two STS models: **(A)** in IB115 cell line xenograft after 3 weeks of treatment and **(B)** in PDX model JR588. After 3 weeks of treatment, tumour volume was measured 10 days after the end of treatment. In vitro, there is a synergistic effect with the two drugs for IB115 and JR588 (data not shown) **(C)** yH2AX IHC analysis in PDX model for vehicle, each drug used as single agent or in combination. **(D)** The graph represent the two values of IHC score. Slides were digitalized at 20X on a PhenoImager HT (Akoya) and tissues were analyzed using Inform software v2.6 (Akoya), an IHC score for yH2AX positivity (2-bin) was defined based a 0.25 positivity threshold for each condition

Finally, we assessed the impact of combined AZD6738/AZD0156 treatment in two in vivo models representing the two most frequent histological sarcoma subtypes: dedifferentiated liposarcoma (IB115) and undifferentiated pleomorphic sarcoma (JR588). Combined treatment reduced tumour growth, whereas single-agent treatments provided little benefit over the control (Fig. 2). Moreover, assessing pharmacodynamics biomarkers for DNA damage, revealed a significant increase for γH2AX staining with the combination treatment in comparison with each drug used as a single agent (Fig. 2).

Exploiting synthetic lethality is a recurring theme in the field of anti-cancer drug development efforts. CRISPR-Cas9 knockout screens represent invaluable tools to identify genes whose loss sensitize tumor cells to a specific inhibitor. To identify biomarkers associated with sensitivity to AZD6738, Wang et al. used a CRISPR screen approach in the 293 A, HCT116, MCF10A cell lines [8]. As observed in our study, the authors identified an enrichment in DDR repair pathways. They also found that RNASEH2 loss sensitized tumors cells AZD6739, implying there is synthetic lethality between RNASEH2 activity and ATR inhibition. Hustedt et al. also performed a genome-wide CRISPR-Cas9 screening to identify genes whose loss was associated with sensitivity to two ATR inhibitors; VE-821 or AZD6738 [9]. They found that POLE3/POLE4 genes loss was associated with increased sensitivity to ATR inhibitors in HCT116 and HeLa cells, as well as in a p53-mutated clone of RPE1 hTERT, which are telomerase-immortalized retinal pigment epithelial cells. To the best of our knowledge, we report here the first study using a genome-wide CRISPR screening techniques to identify genes associated with sensitivity to ATR inhibition in sarcoma models and particularly in most frequent histotypes, and we confirmed their therapeutically interest in in vitro and in vivo models.

As observed in our previous study, the effects of ATR inhibition were modest both in vitro and in vivo [6]. However, by undertaking CRISPR screens, we identified THRAP3 loss as a key determinant of sensitivity to ATR inhibition. THRAP3 is a key mediator of resistance to DNA damage and is crucial for efficient DNA repair and cellular survival. Pre-clinical studies have shown that a transient depletion of THRAP3 induces sensitivity to DNA damaging agents due to deficient processing of transcripts encoding the ATM kinase [7, 10]. ATR and ATM have been reported to share a synthetic lethal relationship in several tumor types [9, 11]. Our study confirms that pharmacological inhibition of ATM sensitizes several subytpes of STS cells (including most frequent histologies), to ATR inhibition whatever their initial level of sensitivity to ATR targeting. Even if the anti-tumor activity of combined ATR and ATM inhibition may differ according to histological subtype, our findings have important clinical implications and suggest this approach deserve further investigation in patients with STS.

## Electronic supplementary material

Below is the link to the electronic supplementary material.

Supplementary Material 1: Materials and Methods, and Supplementary data

## Data Availability

Data are available upon request to the corresponding author.

## List of abbreviations

**ATM**: ataxia-telangiectasia mutated
**ATR**: ataxia telangiectasia and Rad3-related
**DDR**: DNA Damage Response
**STS**: soft-tissue sarcomas
