# Supplementary material for: Genome-wide CRISPR/Cas9 library screening identified ATM signaling network genes as critical drivers for resistance to ATR inhibition in soft-tissue sarcomas: synthetic lethality and therapeutic implications
Source: Exp Hematol Oncol. 2023 May 31;12:51. doi: 10.1186/s40164-023-00416-z (PMC10234020; doi:10.1186/s40164-023-00416-z)
Supplement: Supplementary file 1 — Supplementary Material 1: Materials and Methods, and Supplementary data [file 40164_2023_416_MOESM1_ESM.docx]

**MATHERIAL AND METHODS**

***Cells and cell culture.*** All cell lines we used derived from human surgical specimens of STS after obtaining written informed patient consent and Institut Bergonié institutional review board approval. Each cell line was characterized by array comparative genomic hybridization for every 10 replicates to verify that its genomic profile was still representative of the originating tumor sample. We chose 7 cell lines representative of the most frequent histologies of STS (Supplementary Table 1). Cells were grown in RPMI (Roswell Park Memorial Institute) medium 1640 (Sigma Life Technologies, Saint Louis, MO) in the presence of 10% fetal calf serum (Dutscher, France) in flasks. Cells were maintained at 37°C in a humidified atmosphere containing 5% CO2, and they were routinely passaged every 4-5 days.

***Drugs.*** The ATR inhibitor (ATRi) AZD6738 and the ATM inhibitor (ATMi) AZD0156 were supplied by Astrazeneca as powder and diluted in DMSO for final solutions of 30 µM and 10 µM respectively. The aliquots of the stock solutions were stored at -20°C.

***Cell viability assays.*** The 7 cell lines were screened for sensitivity to the ATRi alone. For the antiproliferative and cytotoxic assays we used Cytation™ 3 technology (Colmar, France), a cell imaging multi­mode microplate reader that combines automated digital microscopy and conventional microplate detection. Cells were seeded in 384-well plates according to the rate of cell growth (800 cells/well for the IB114, IB115, IB128; 1000 cells/well for the 93T449; 1500 cells/well for the IB111, IB112 and IB136). After 24 hours, cells were exposed to a range of drug concentration from 0.125 to 64 µM for each drug for 72 hours, to determine the half inhibitory concentration (IC_50_). Cells were then marked with a PBS 1x solution containing 3 µg/ml of propidium iodide (PI), a not membrane-permeable red fluorochrome which stains DNA of necrotic/apoptotic cells, 6 µM Syto 24 solution, a cell-permeant green fluorescent nucleic acid stain, which stains both live and dead cells, and 60 µg/ml of RNase A, for each plate. Cells were exposed to the fluorochromes for 30 min in the dark at 37°C in a humidified atmosphere containing 5% CO_2_. Quantitative fluorescence and cell imaging were performed with Cytation™ 3 at λ = 617 nm for PI (λ excitation = 538 nm) and 521 nm for Syto 24 (λ excitation = 495 nm). The half maximal inhibitory concentration (IC_50_) was calculated with GraphPad Prism software (GraphPad Software, La Jolla, CA, USA) using a non-linear regression analysis.

In a second time, for the ATRi and ATMi combination essay, the 7 cell lines were screened for sensitivity to the ATRi alone, ATMi alone and in combination. For the combination, the same range of concentration of the two drugs was used. All steps followed the same protocol than the single drug essay.

**Cell Viability assay: MTT** ATRi effects on cell viability were investigated using the MTT assay [3-(4,5-dimethylthiazol-2-yl)-2,5-diphenyl tetrazolium bromide, Sigma-Aldrich Chimie,Saint-Quentin-Fallavier, France] as an indicator of metabolically active cells. Known number (3000 cells) of IB112-cas 9 or IB112-X-knockdown cell lines were transferred into 96-well plates incubated for 24 h before addition of test compound. Cells were then exposed for 72 h at 37°C to an increasing concentration range of ATRi. MTT at the final concentration of 0.5mg/ml was added and following incubation for 3h, Formazan crystals were dissolved in DMSO. Absorbance of the colored solution was measured on a microplate-photometer (Molecular Device ABS Plus) using a test wavelength of 570 nm and a reference wavelength of 630nm. The concentration of substance required for 50% growth inhibition (IC_50_) was estimated with the GraphPad Prism software.

**CRISPR screen** For CRISPR screen, the human knockout pooled library GeCKO V2 (Addgene 1000000048) was used. This library is comprised of 123411 sgRNAs that target 19050 genes). 240 millions of IB112-cas 9 cells were infected at a multiplicity of infection (MOI) of 0.3 to obtain a 500X coverage and were selected during 3 days at 1µg/mL of Puromycin. For ATR resistance screen, 30x10^6^ cells were treated by DMSO and 30x10^6^ cells were treated by 8µM of ATRi. Cells were treated during 72h and then collected for DNA extraction. We performed a negative selection, by interesting to knockout cells sensitive to the treatment (basically, to guides we lost after treatment).Comparison of treated cells versus, control survival was around 20%.

**Sequencing and analysis of CRISPR screen** Genomic DNA was extracted with DNeasy Blood and Tissue kit (Qiagen ID: 69504) according to the manufacturer’s protocol. The totality of genomic DNA was sequenced by Nucleus Biotech. SgRNA were amplified and next generation sequencing (NGS) was performed on the Illumina NextSeq targeting 60 millions of reads. Results were analyzed with Mageck, Riger and Crispr cloud softwares (Jeong et al. 2017). This screen allowed us to identify the genes, the inactivation of which conferred lethality with ATR inhibition(meaning the genes that was inactivated in cells we lost with the treatement).

Infection assay : In order to knock down gene of interest, IB112 cells were infected at a multiplicity of infection (MOI) of 5 ,or 10 with the sgRNA of the GecKo bank who has given the best result after the screening (ref : THRAP3 CRISPR guide RNA1 _ pLentiGuide – Puro, Genscript SC1678)

***Apoptosis assays.*** For apoptosis assessment, 2x10^5^ cells were seeded in 6-well plates. After 24 hours, cells were treated with ATRi and ATMi alone and in combination for 72h. The doses were chosen considering the IC_50_ obtained with the cell viability assays. For each line, the dose selected was the one which induced up to 80% of inhibition of proliferation , which is in the combination treatment (4 µM for the IB115, 2 µM for the IB114, 16 µM for the IB112, and 16 µM for the IB136). For positive control, one well of each experiment was treated with 2 µM of Staurosporine for 6 hours. Then, the supernatant was collected; cells were washed twice with PBS, trypsinized, collected and centrifuged at 1500 rpm for 5 minutes at 20°C. The supernatant was discarded, and cells were marked with the “Biolegend™ FITC Annexin V Apoptosis Detection Kit with PI” according to the manufacturer’s protocol. This allowed us to distinguish Annexin V-positive cells in early apoptosis from Annexin V- and PI-positive cells in late apoptosis. Cells were analyzed by flow cytometry using FL1 for Annexin V and FL2 for PI and analyzed using Cell Quest Pro software (BD Biosciences, San Jose, CA, USA). Flow cytometry (FACScan; BD Biosciences) data were analyzed with FlowJo v.7.6.3 software.

***Cell cycle analyses.*** Cell cycle distribution was studied by examining DNA content using flow cytometry and analyzed using Cell Quest Pro software (BD Biosciences, San Jose, CA, USA). 2x10^5^ cells/well were seeded in 6-well plates, using two wells for each condition. As soon as plated, the cells were starved for 24 hours using serum-free medium, and then treated for 48 hours with ATRi and ATMi alone and in combination. The chosen doses were the same used for the apoptosis assays. At the end of treatment, the supernatant was aspirated, and cells were washed with PBS, trypsinized, collected, and centrifuged at 5000 rpm for 5 minutes at 20°C and washed with PBS twice. The cells were then fixed with 70% ethanol at -20°C for at least 3 hours. Following ethanol removal, the cells were washed twice with PBS and centrifuged at 5000 rpm for 5 minutes. Next, 300 µl of a PBS 1x solution containing 50 µg/ml of PI and 5 µg/ml of RNase were added to the cell pellets. After 30 minutes of incubation in the dark at room temperature, they were analyzed by FACS. The data were analyzed with FlowJo v.7.6.3 software, and the results were expressed in terms of percentage of cells in each phase of cycle.

***Western Blot.*** Control and 24 hours-treated cells were harvested in the radio-immunoprecipitation assay (RIPA) lysis buffer (NaCl 150mM, Tris pH 7.5 50mM, NP40 1%), containing a mix of protease inhibitors (Roche®) and phosphatase inhibitors (NaO 1mM and NaF 1mM). The lysate was centrifuged (13000 rpm, 10 min, 4°C), and the supernatant was stored at -20°C. Protein concentration was measured through a colorimetric dosage, using the “DC™ Protein Assay” kit by BioRad. The optical density of the samples was compared with that one of a BSA standard range through. Protein samples were denatured by the Laemmli Buffer (TrisHCL 83 mM pH 6.8, glycerol 35%, SDS 4%, beta-Mercaptoethanol 1%, blue bromophenol 0.04%) and boiled at 95° for 5 minutes. As ATM is a 350 kDa protein, a low percentage gel was necessary for the detection of such a large protein. Then, for pATM/ATM expression analyses, equal amounts of total protein (80 µg) were electrophoresed on 4-20% sodium dodecyl sulfate (SDS) polyacrylamide precast gels and transferred onto polyvinylidene difluoride (PVDF) membranes with a rapid transfer, by using iBlot^®^ 2 Dry Blotting System (Life technologies™), with the pre-programmed template for large proteins P0 (20V for 1min, 23V for 4min, 25V for 2min). CHK1 is a 56 kDa protein. Therefore, for pCHK1/CHK1 expression analyses, equal amounts of total protein (50 µg) were electrophoresed on 12% sodium dodecyl sulfate (SDS) polyacrylamide gels and transferred onto polyvinylidene difluoride (PVDF) membranes with a semi-liquid transfer for 60 min at 100V. The blots were probed overnight at 4°C in either 5% BSA or milk in PBS 0.1% Tween-20 with the primary antibody (all used primary antibodies are listed in the Supplementary Table 2), and then with a horseradish peroxidase-conjugated secondary antibody at room temperature for 1h. For all blots, the phosphorylated proteins were the first proteins detected on each membrane. After stripping in a glycine buffer 0.15M pH2, the respective total proteins and the housekeeping proteins were detected. For the loading control, Vinculin was the housekeeping protein used for the 4-20% gels, due to its high molecular weight (116 kDa); actin was the one used for the 12% gels (42 kDa). Bound antibodies were visualized on Fusion Fx7 imaging system (Fisher Bioblock Scientific, Waltham, MA, USA) using the Immobilon™ Western enhanced chemiluminescence detection kit (Millipore Corporation, Billerica, MA, USA). The resulting bands were analyzed and quantified using ImageJ^®^ 1.49g software (National Institutes of Health, Bethesda, MD, USA).

For the analyses, the ratio between the phosphorylated and the total protein was used for measuring pATM variations. On the other hand, to analyze pCHK1 levels, the same ratio was not representative, as total CHK1 levels changed in relation to treatments. Then, to check the real consequences of drugs on the pathways, total CHK1 was normalized on actin levels, and the final ratio was pCHK1/(CHK1/actin)

For CRISPR screen experiments and validation we have performed Western blotting analysis for evaluation of cas9 expression (cas9 antibody 1/1000, Cell signaling) in IB112 cells after infection and we have controlled the extinction of protein THRAP3, with antibody from Bethyl (1/1000).

***In vivo experiments.*** All animal experiments were performed with the approval of the institutional animal use and care committee under project license APAFIS#8415-2017010211442345 (University of Bordeaux). This study followed the French and European Union guidelines for animal experimentation (RD 1201/05, RD 53/2013 and 86/609/CEE, respectively). Induction of tumor xenografts was performed by subcutaneous implantation of UPS tumor fragment (PDX) or injection of IB115 cells (3x10^6^ cells/200µL) into the right flank of the RagƔ2C-/- mice (n = 10 per group). Once palpable, tumor volumes were calculated using the following formula: length × width2/2. Once the average size of the tumors was 100 mm3, animals were treated with the ATRi and the ATMi via oral gavage. The mice were randomized into four groups: vehicle, ATRi alone (50mg/kg, oral gavage 5 times per week), ATMi alone (5mg/kg, oral gavage 3 times per week), and both drugs (ATRi and ATMi, 50mg/kg, oral gavage 5 times per week and 5mg/kg, oral gavage 3 times per week respectively). Mice in each group were treated for 3 weeks, after which treatment was stopped and tumors were measured every 2–3 days with calipers and the diameters were recorded. Mice were euthanized when the tumor volume reached 2000 mm3. Tumor progression was analyzed with GraphPad Prism software, and Kaplan–Meier curve analysis was used to compare the overall survival. Log-rank (Mantel–Cox) tests were used to compare Kaplan–Meier curves, and p-values of 0.05 and below were considered statistically significant. For the JR588 PDX model we sacrificed in each group 1 mouse per week of treatment to make staining for phosphorylated proteins, notably γH2AX to verify that the effect we see was due to the mitotic catastrophe.

***Immunohistochemistry (IHC)*** IHC on paraffin embedded tumors was performed using the Ultraview/VENTANA detection system, Phospho-histone H2ax was stained with rabbit monoclonal antibody (OZYME, ref 9718), DAB (3, 3’-Diaminobenzidine) was used to detect bound primary antibodies. Dako Hematoxylin was used to counter stain nuclei. Images were acquired on a Panoramic 250 Flash III Digital Slide Scanner (3DHISTECH^TM^). IHC for Phospho-H2ax expression on cell lines and mice tumors (P-H2ax mAb-5, 1/400, 56 min) was performed with Ultraview/Ventana system after a CC1 light (Cell conditioning 1 light) process (96°C, 36 min).

***Statistical analyses.*** All experiments were reproduced two or three times to confirm their reproducibility. The statistical analyses were done with GraphPad software. The results were expressed as mean +/- SEM (standard error of the mean). The cell viability assays were evaluated by non-linear regression analysis (sigmoid dose response curve, variable slope). The apoptosis and cell cycle assays were evaluated by the variance analysis (ANOVA test). Correlation analyses were conducted using Person R test; significant differences are indicated as *p<0.05, **p<0.01 and ***p<0.001. The accepted alpha error was 5%.

**SUPPLEMENTARY TABLES**

| **Supplementary Table 1.** | |
| --- | --- |
| **Cell lines** | **Histological subtype** |
| IB128 | Extra-skeletal osteosarcoma |
| IB111 | Dedifferentiated liposarcoma |
| IB114 | Myxofibrosarcoma |
| 93T449 | Well differentiated liposarcoma |
| IB115 | Dedifferentiated liposarcoma |
| IB136 | Leiomyosarcoma |
| IB112 | Leiomyosarcoma |
| JR588 | Undifferentiated pleomorphic sarcoma |

| **Supplementary Table 2.** | | |
| --- | --- | --- |
| **Antibody** | **Pharma** | **Dilution** |
| Anti-CHK1 phospho296 | Abcam, ab79758 | 1/1000 in 5% BSA in PBS 0,1% Tween-20 |
| Anti-CHK1 | MBL, K0086-3 | 1/1000 in 5% BSA in PBS 0,1% Tween-20 |
| Anti-ATM phosphoS1981 | Abcam, ab81292 | 1/1000 in 5% BSA in PBS 0,1% Tween-20 |
| Anti-ATM | Abcam, ab32420 | 1/5000 in 5% milk in PBS 0,1% Tween-20 |
| Anti-Vinculin | Sigma, V9131 | 1/400 in 5% milk in PBS 0,1% Tween-20 |
| Anti-Actin | Sigma, A5441 | 1/5000 in 5% BSA in PBS 0,1% Tween-20 |
| Anti-Thrap3 | Bethyl, A300956AT | 1/1000 in 5% BSA in PBS 0,1% Tween-20 |

**SUPPLEMENTARY FIGURES**

**
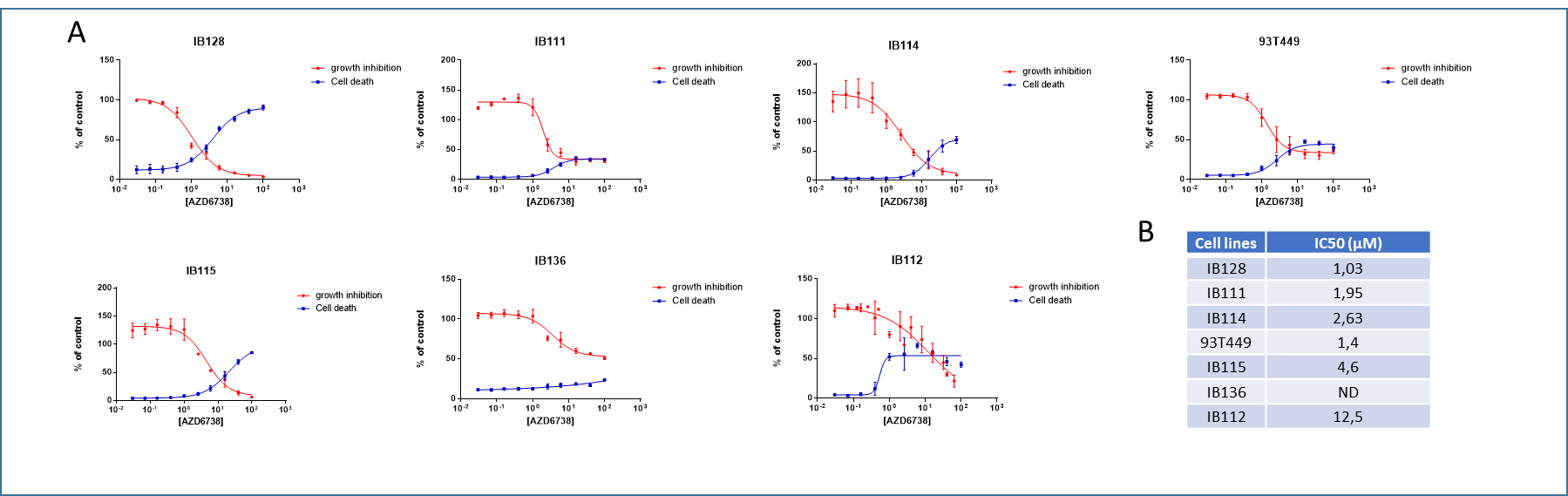
**

**Supplementary Figure 1: Effect of AZD6738 on growth inhibition and cell death in 7 STS cell lines.** A) Efficacy of AZD6738 was measured with increasing doses, and IC50 (µM) was calculated. The two Leiomyosarcomas IB112 and IB136 are resistant to the AZD6738 with IC50 at 12.5 µM and not determined respectively. B) Table of IC50 for AZD6738 for the 7 STS cell lines.

**
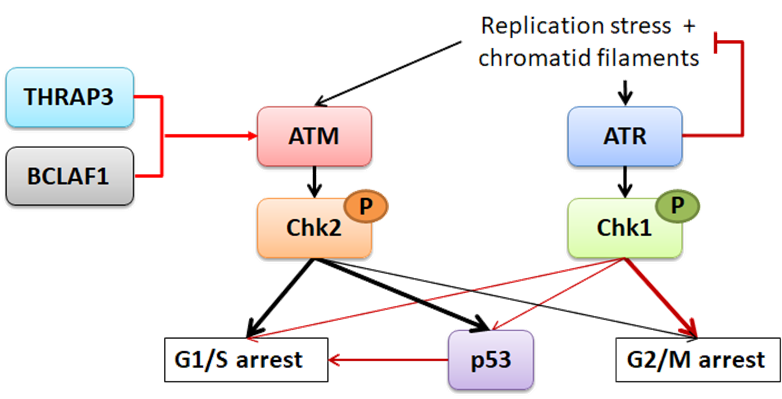
**

**Supplementary Figure 2.** Thrap3 and Bclaf1 are involved in maturation and export of transcript encoding ATM. Its depletion results in active ATM kinase deficiency. Even more, ATM is a parallel pathway of ATR, taking over when ATR pathway is inhibited. Finally, Thrap3 could be responsible for ATR resistance through ATM.


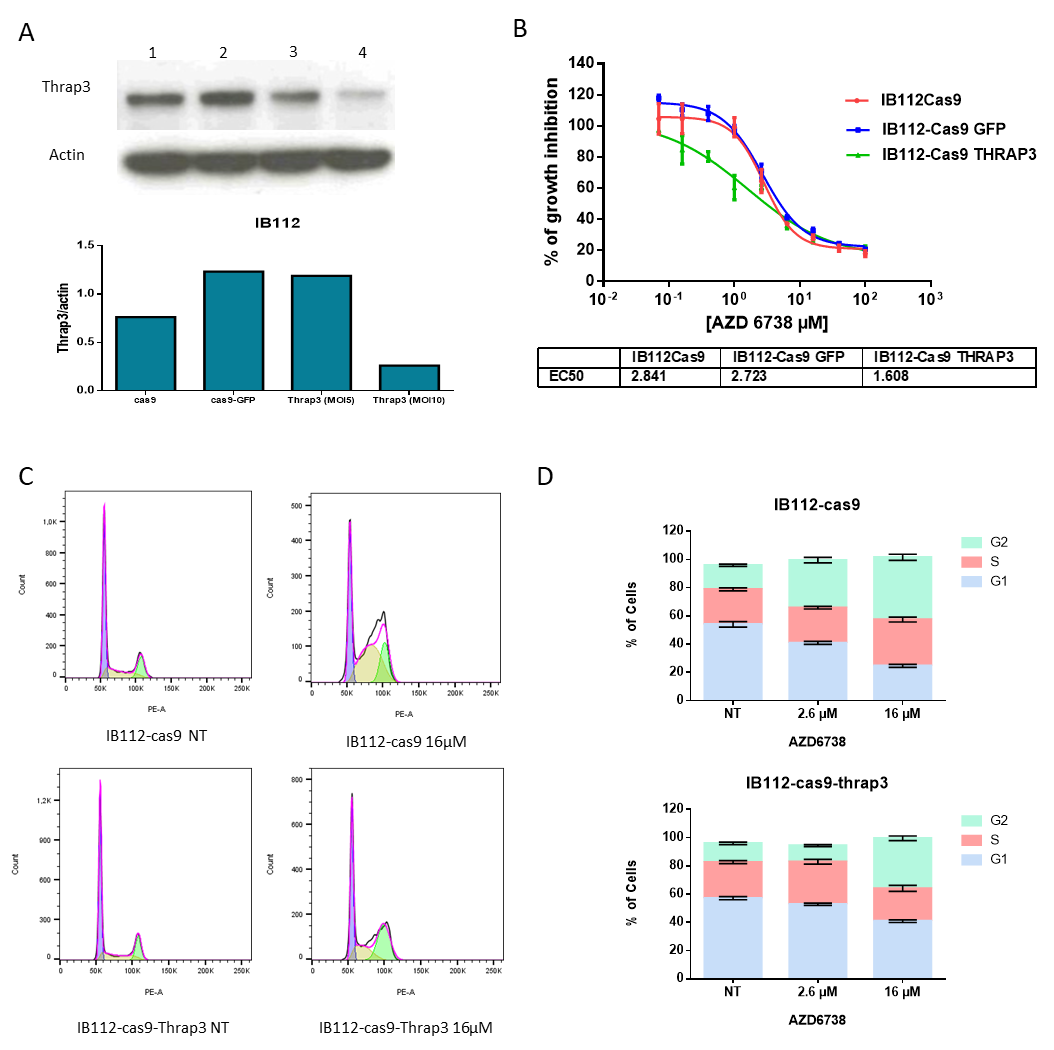


**Supplementary Figure 3:** Validation of the potential role of the *THRAP3* gene in the resistance of IB112 cells to ATR inhibition. A) Western blot and quantification of Thrap3 and Actinin (1) IB112-cas9, (2) IB112-cas 9-GFP, (3) IB112-cas9-Thrap3 (MOI = 5) and (4) IB112-cas9-Thrap3 (MOI = 10). B) Cytotoxicity analysis by MTT in control cells (IB112-cas9 and IB112-cas9-GFP) and Thrap3 silenced cells, the IC_50_ are 2.8, 2.7 and 1.6 µM respectively. C) Cell-cycle profile after 48 h of treatment with the ATRi analysed by PI incorporation and flow cytometry in the IB112-cas9 and IB112-cas9-thrap3 cell line untreated or treated with 16µM of AZD6738; on the FACS plots: violet represents the phase G0/G1, yellow phase S and green phase G2/M. D) Cell-cycle distribution was calculated from the flow cytogram.


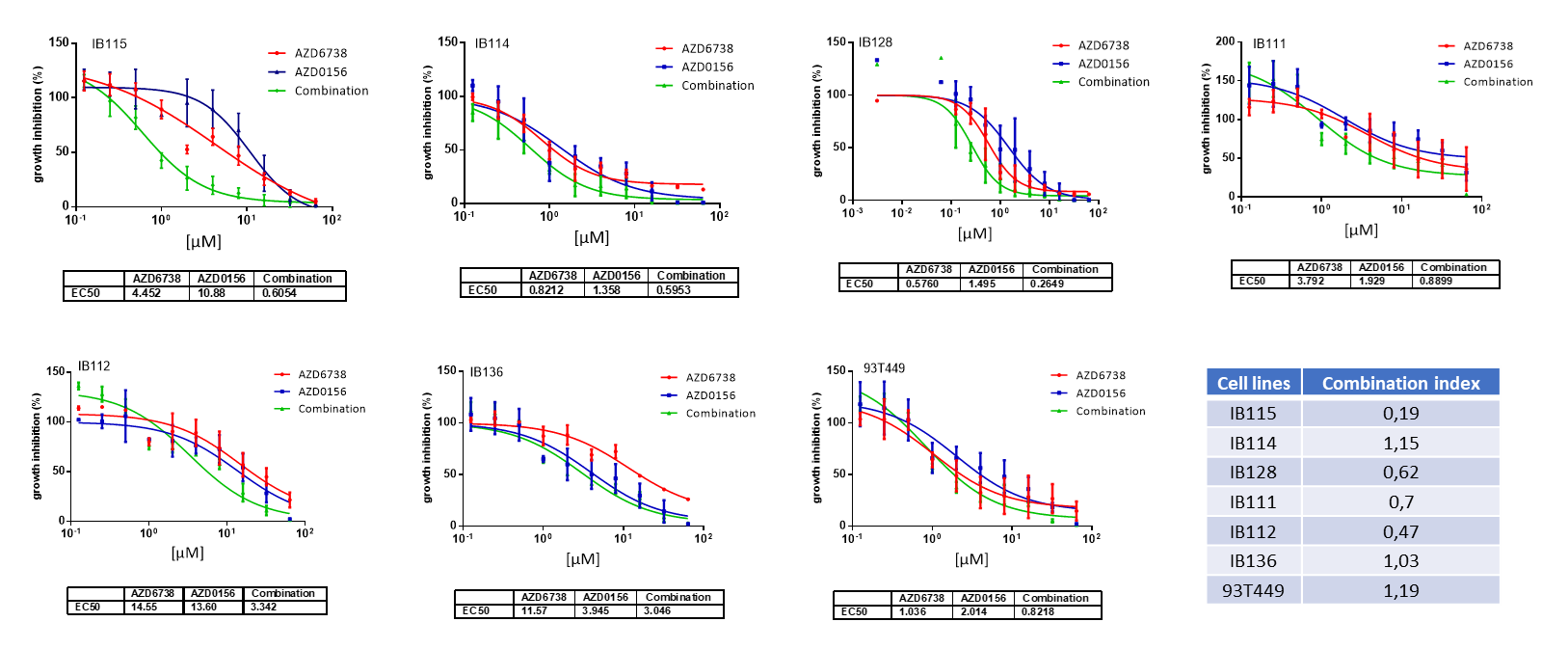


**Supplementary Figure 4:** **Effect of ATR inhibitor, ATM inhibitor, and combination treatment in STS cell lines.** Efficacy of ATR inhibitor (AZD6738), ATM inhibitor (AZD0156), and combination treatment in STS cell lines: IB115, IB114, IB128, IB111, 93T449, IB112, IB136 treated with an increasing range of doses, from 0 to 64 µM for 72h. IC_50_ (µM) was calculated using a non-linear regression analysis. Combination index (CI) < 1, CI = 1, and CI > 1 indicates synergistic, additive, and antagonistic effects, respectively. Experiments reproduced three times.


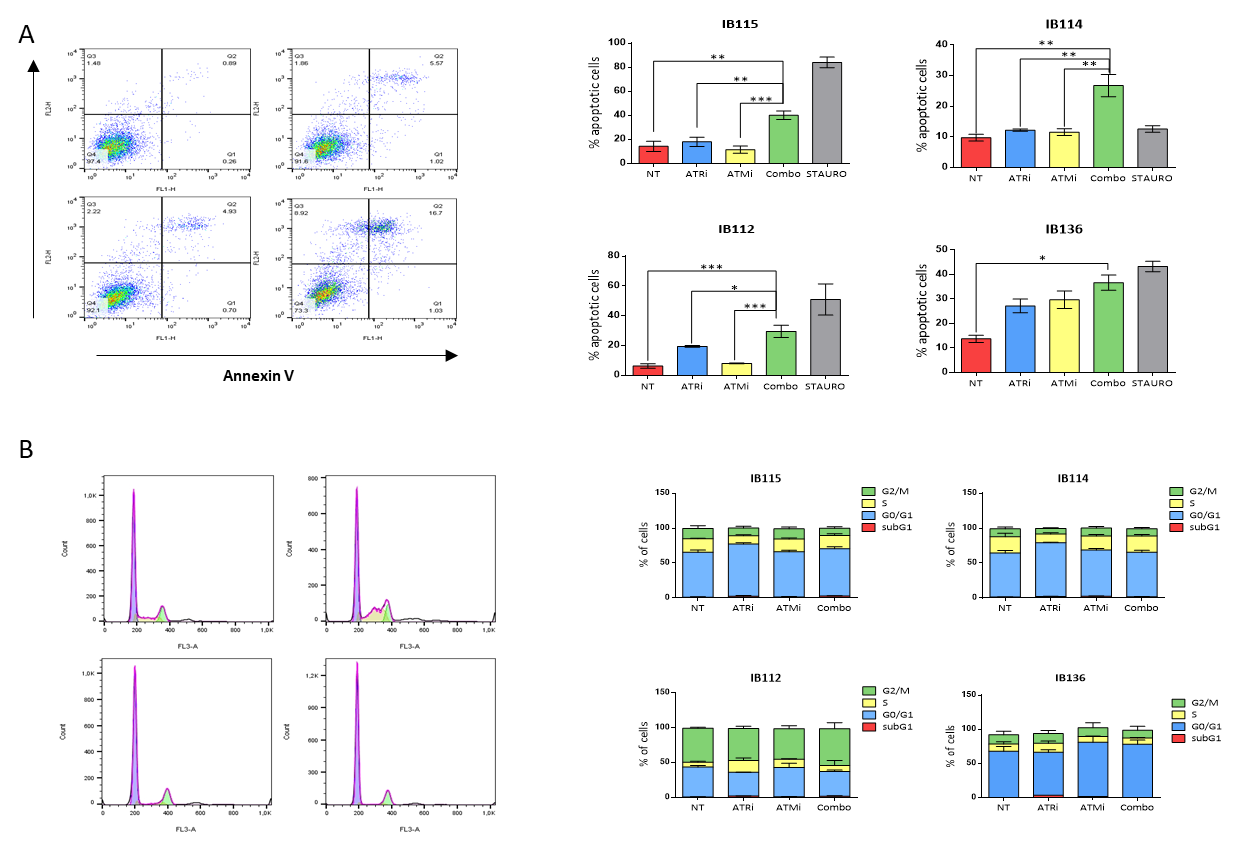


**Supplementary Figure 5:** **A)** **Cytotoxicity of ATR inhibitor, ATM inhibitor, and combination treatment.** Apoptosis induced by ATR inhibitor (AZD6738), ATM inhibitor (AZD0156), and combination treatment in STS cell lines, treated for 72h, at the dose inhibiting up to 80% of the proliferation in the most efficient setting (combination treatment): 4µM for the IB115, 2µM for the IB114, 1µM for the IB128, 16µM for the IB112, and 16µM for the IB136. Ctrl= staurosporine positive control. The flow plot is a representative plot for the IB112 cells. **B) ATR inhibitor, ATM inhibitor, and combination treatment effect on cell cycle.** Effect on cell cycle of ATR inhibitor (AZD6738), ATM inhibitor (AZD0156), and combination treatment in a sample of synergistic and additive STS cell lines, treated for 48h, at the dose inhibiting at most 80% the proliferation in the most efficient setting (combination treatment): 4µM for the IB115, 2µM for the IB114, 1µM for the IB128, 16µM for the IB112, and 16µM for the IB136. * p ≤0.05, ** p ≤0.01, *** p ≤0.001. Experiments reproduced three times. **On the FACS plots: violet represents the phase G0/G1, yellow phase S and green phase G2/M.**


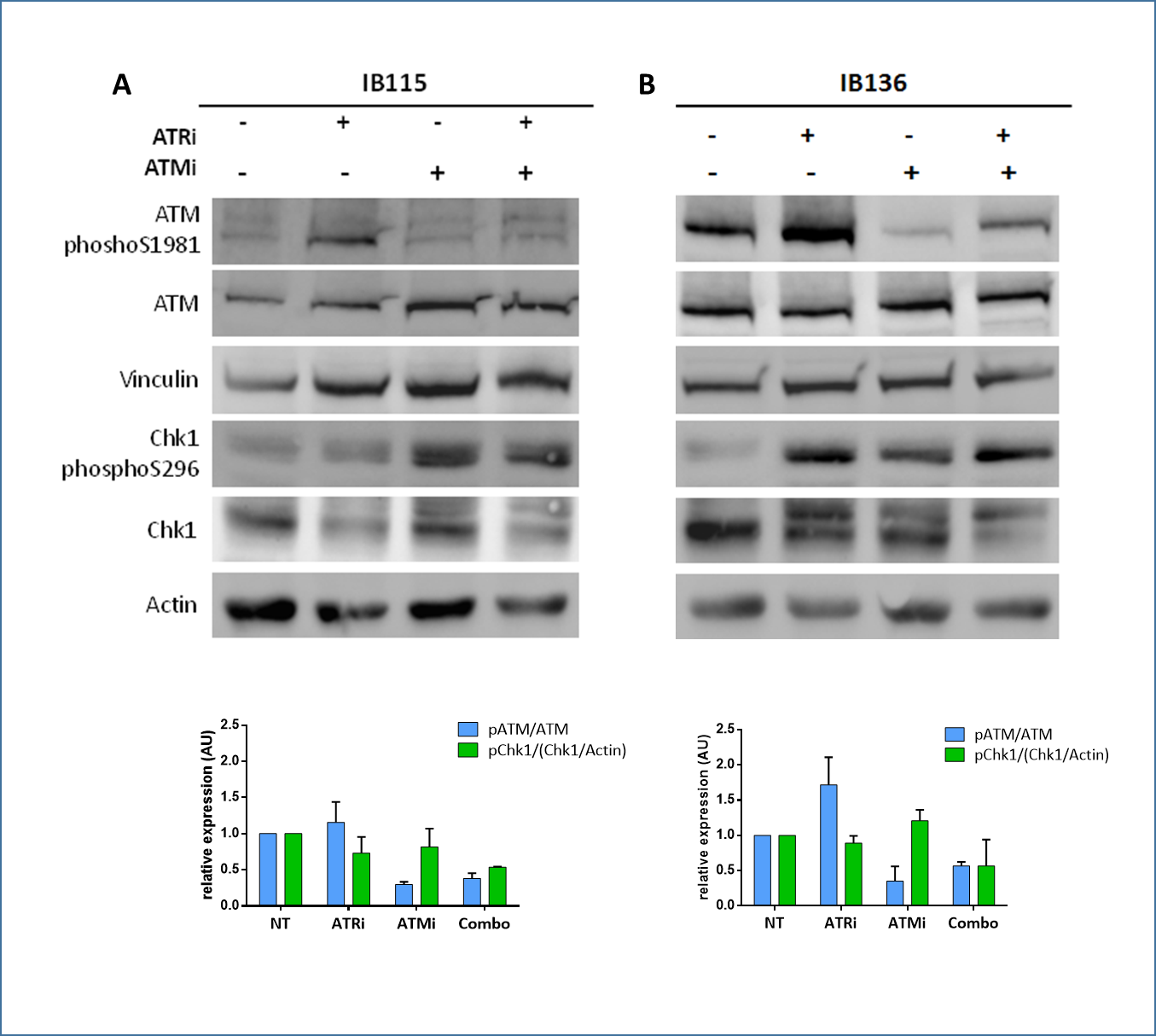


**Supplementary Figure 6:** **ATR and ATM pathways differently respond to the ATRi and ATMi in STS cell lines. (A)** For pATM/ATM detection 80 µg of proteins for each line were loaded in a 4-20% SDS polyacrylamide precast gel and transferred on a PVDF membrane after 120min of migration at 150V. For pChk1/Chk1 immunoblotting, 50 µg of proteins for each line were loaded in a 12% SDS polyacrylamide gel and transferred on a PVDF membrane after 180min of migration at 120V. Experiments reproduced twice. **(B)** For pATM quantification, the ratio pATM/ATM was calculated for each condition. For pChk1 quantification, pChk1(Chk1/Actin) ratio was calculated for each condition, to normalize the total Chk1 variations related to each treatment. Experiments were reproduced 2 times.
